# Understanding the Prevalence and Geographic Heterogeneity of SARS-CoV-2 Infection: Findings of the First Serosurvey in Uttar Pradesh, India

**DOI:** 10.1007/s44197-021-00012-6

**Published:** 2021-10-26

**Authors:** Vasanthakumar Namasivayam, Amita Jain, Vikasendu Agrawal, Ravi Prakash, Bidyadhar Dehury, Marissa Becker, James Blanchard, Shajy Isac, Amit Mohan Prasad

**Affiliations:** 1 Department of Community Health Sciences, Institute for Global Public Health, University of Manitoba, R070 Med Rehab Bldg, 771 McDermot Avenue, Winnipeg, MB R3E 0T6 Canada; 2 India Health Action Trust, New Delhi/Lucknow, India; 3 King George’s Medical University (KGMU), Lucknow, India; 4 Government of Uttar Pradesh, Lucknow, India

**Keywords:** COVID-19, SARS-CoV-2, Seroprevalence, Heterogeneity, Uttar Pradesh, India

## Abstract

Population-based serological antibody test for SARS-CoV-2 infection helps in estimating the exposure in the community. We present the findings of the first district representative seroepidemiological survey conducted between 4 and 10 September 2020 among the population aged 5 years and above in the state of Uttar Pradesh, India. Multi-stage cluster sampling was used to select participants from 495 primary sampling units (villages in rural areas and wards in urban areas) across 11 selected districts to provide district-level seroprevalence disaggregated by place of residence (rural/urban), age (5–17 years/aged 18 +) and gender. A venous blood sample was collected to determine seroprevalence. Of 16,012 individuals enrolled in the study, 22.2% [95% CI 21.5–22.9] equating to about 10.4 million population in 11 districts were already exposed to SARS-CoV-2 infection by mid-September 2020. The overall seroprevalence was significantly higher in urban areas (30.6%, 95% CI 29.4–31.7) compared to rural areas (14.7%, 95% CI 13.9–15.6), and among aged 18 + years (23.2%, 95% CI 22.4–24.0) compared to aged 5–17 years (18.4%, 95% CI 17.0–19.9). No differences were observed by gender. Individuals exposed to a COVID confirmed case or residing in a COVID containment zone had higher seroprevalence (34.5% and 26.0%, respectively). There was also a wide variation (10.7–33.0%) in seropositivity across 11 districts indicating that population exposed to COVID was not uniform at the time of the study. Since about 78% of the population (36.5 million) in these districts were still susceptible to infection, public health measures remain essential to reduce further spread.

## Introduction

The World Health Organisation (WHO) declared severe acute respiratory syndrome coronavirus 2 (SARS-CoV-2), or the novel coronavirus, to be a public health emergency of international concern in March of 2020 [1]. By the end of 2020, over 81 million people worldwide had been diagnosed with COVID-19, and 1.8 million had died [2]. Available evidence suggests that reported figures considerably understate the extent of the spread of the virus [3]. With over 80% of the total cases estimated to be asymptomatic, it has been widely acknowledged that laboratory-based surveillance systems have not been able to capture the total magnitude of the virus spread. As such, the prevalence rates of COVID-19 within the population remain largely underestimated [3, 4].

Serological antibody testing of the population becomes important as it can enable improved understanding and can help derive correct estimates of the true extent of the disease spread [5]. An antibody-based testing strategy has been recommended by the WHO for overcoming the limitation of selective testing of facility-based surveillance systems and to better estimate the prevalence of both asymptomatic and mildly symptomatic cases in the community [6]. Numerous countries heavily afflicted by the pandemic have conducted large-scale serosurveys using serologic antibody tests to help quantify the actual burden of COVID-19 [7]. Different methods (rapid tests, lateral flow immunoassays and ELISA-based tests) and strategies of testing (drive-through testing, school-based testing and volunteer screening) have been implemented. Positivity rates in population-based surveys have ranged from 5.0% in Spain to 5.3% in France [8, 9], while the prevalence levels ranged from 14.0% in New York, USA and Gangelt, Germany to 10.8% in Geneva, Switzerland and 0.5% in San Miguel County, USA [4, 10].

Similarly, in India, federal agencies, states and cities have conducted various independent studies to estimate seropositivity levels including the studies conducted by the Indian Council of Medical Research (ICMR), the apex health reaserch institute of India. With 28,000 adults from the 70 districts of the 21 states of India, the first serological study by ICMR revealed a pooled adjusted seropositivity rate of 0.73%, indicating that nearly 6.4 million people had been exposed to COVID-19 by early May 2020 [11]. A subsequent survey by ICMR covering more than 29,000 individuals aged 10 years and above across the country, estimated seropositivity to be 6.6% by the end of September 2020 and 21.4% by mid-December 2020 [12, 13]. This indicated that approximately 280 million people cumulatively had been infected by the virus by the end of 2020 in India. Higher prevalence was reported in urban slums (15.6%) compared to urban non-slums (8.2%) and rural geographies (4.4%). State-level surveys in Punjab (May 2020) and Andhra Pradesh (June 2020) detected SARS-CoV-2 prevalence of 24.2% and 19.7%, respectively [14, 15]. Localised serosurveys found even higher rates of virus exposure. For example, investigations in the country’s major urban locales revealed seropositivity to be as high as 51.3% in Pune city, 22.9% in Delhi and 23.2% in Ahmedabad city [16–18] by July 2020. In Mumbai, positivity rates were observed to be considerably higher among slum residents (54.1%) compared to the non-slum urban population (16.1%) during the same period [19].

In Uttar Pradesh (UP), the most populous state in India with an approximate population of 225 million [20], the number of confirmed cases of COVID-19 by the end of August 2020 stood at 230,414 [21, 22]. While some serosurveys had been conducted in different parts of the country, similar estimates from high-burden districts of UP were largely missed. The national-level serosurvey conducted by ICMR had covered certain districts in UP, yet district-level estimates could not be derived due to the survey design and relatively smaller district-level sample size. Given the expanse of the state and its centrality to the country’s efforts to further contain the spread of COVID-19, understanding the heterogeneity in prevalence levels was required. We implemented a seroprevalence study to understand the epidemiological profile of disease burden by geography (rural/urban and high/low prevalence districts), population sub-groups (age and gender) as well as other high-risk categories (people with known comorbidities, including diabetes, hypertension, immunocompromised conditions and severe acute respiratory illnesses, among others). Findings of the study would have important programmatic relevance in informing the strategy to help mitigate the epidemic locally.

## Materials and Methods

### Study Setting

The Government of UP (GoUP), in collaboration with King George’s Medical University (KGMU), Lucknow, and the Uttar Pradesh Technical Support Unit (UPTSU) of the University of Manitoba (UoM) and India Health Action Trust (IHAT), conducted its first district-level seroepidemiological study in 11 districts of UP to estimate the prevalence of infection among the population aged 5 years and above. These districts were identified by the state government based on the relatively high burden of cases reported from these geographies and were among those not covered in the national serosurvey conducted by ICMR. These 11 districts together contributed 20% of the total population in the state and 40% of the total COVID-positive cases at the time the survey was conducted.

### Sampling Design and Participants

A multi-stage cluster sampling approach was used for this study implemented in rural and urban areas of 11 districts of the state between September 4th and 10th, 2020. The study estimated 1440 samples assuming a three per cent seropositivity rate for COVID-19 for each of the selected districts, with a precision rate of 1.5%, 95% Confidence Interval (CI), design effect of 2.5 and an assumption of 10% non-response rate (including sample wastage). This amounted to an estimated 15,840 respondents, of which 8928 and 6912 were allocated to rural and urban geographies, respectively. Within each of the selected districts, simple random sampling was used to select 45 Primary Sampling Units (PSU), which constituted census villages in rural areas and wards in urban areas. Altogether, it resulted in 495 PSUs across 11 districts. The catalogue of villages and wards from the 2011 Census was used as the sampling frame. As per the 2011 Census, the rural:urban population proportion of Uttar Pradesh was 78:22 percent. For the 11 districts in which the seroprevalence survey was conducted, the proportion of rural:urban population was 55:45 percent. Accordingly, the number of PSUs within each district was assigned to reflect the respective district’s rural–urban population composition. To ensure representativeness of the samples collected from each of the selected PSUs, we divided each PSU into 4 segments and selected an equal number of samples from each of the 4 segments. By selecting 8 samples from each of the 4 segments within a PSU, we were able to collect 32 samples which would provide adequate samples to calculate the seroprevalence stratified by age and gender at the district level. Thus, the district-wise sample size was allocated as 32 samples across 45 PSUs to achieve 1440 samples per district. The first household within each segment was selected randomly and the remaining 7 households were sequentially selected thereafter. Of these 8 households, the initial 6 households were chosen for the selection of adult individuals (3 men and 3 women), while children aged 5–17 were selected from the remaining 2 households. Only one individual was randomly selected per household. A framework depicting the sample selection process is provided in Fig. 3.

### Data Collection Process

Following an informed written consent, selected participants answered a brief questionnaire that included their socio-demographic profile, history of symptoms compatible with COVID-19 (fever, sore throat, cough, breathlessness), mobility within and outside the community, contact with confirmed COVID-19 cases, history of comorbidities; and had a venous blood sample collected for subsequent laboratory analysis to detect antibodies against SARS-CoV-2.

A total of 110 study teams were constituted, with ten teams being formed in each district. Each study team composed of a Medical Officer (MO), Lab Technician (LT), Community Mobiliser and an interviewer. The UPTSU was involved in survey design, study tool development, data collection monitoring, protocol development, training of investigators as well as data analysis. A data entry module was also developed by UPTSU using Open Data Kits (ODK) to ensure real-time data collection and recording. KGMU was responsible for specimen processing and testing as well as result entry on a web-based portal.

After obtaining written consent, venous blood sample and behavioural data were collected by the health department of GoUP using a central camp approach, wherein selected participants as per study design were mobilized to the camp for interview and blood sample collection. The camp was situated at a central or convenient place within each PSU for easy accessibility. The LT was responsible for blood specimen collection whereas the interviewer collected behavioural information. All the interviews were conducted in a private space to maintain participants’ confidentiality. The MO was in-charge of overseeing the entire data collection process and ensuring adherence to the study protocols. Data collection in a single PSU was completed in 1 day and the blood samples were transported to KGMU, in sealed and sterile packed containers, on the same day following a strict protocol.

### Serology Testing Procedures

The SARS-CoV-2 serological tests were done using ‘COVID KAVACH ELISA (enzyme-linked immunosorbent assay)’ rapid diagnostic tests (RDTs) to detect the presence of IgG antibodies in the blood sample. RDT relies on a lateral flow assay that returns qualitative (positive or negative) results within minutes. The test does not provide quantitative results indicating the amount of antibodies in the specimen. The technology used for this serological testing was approved by ICMR and the manufacturer reported a test specificity of 97.90% and 92.37% sensitivity [23].

During data collection, if an individual was found to be displaying standard symptoms of COVID-19—persistent fever, dry cough, sore throat or breathlessness and/or they were found to be COVID-19 positive, they were still included in the main survey. Symptomatic individuals, and those testing COVID-19-positive on RDT, were provided with appropriate information on the nearest available facilities for COVID-19 testing and were also linked to available healthcare facilities to ensure immediate treatment. Adequate COVID-19 personal protective equipment (PPE) and training were provided by GoUP to the field team to ensure their safety.

### Statistical Analysis

Descriptive analysis was done on the characteristics of the study participants, including place of residence, household size, age, gender, occupation, workplace characteristics, travel history for work or any other purpose, comorbidity status, smoking status, a history of a household or community member who tested positive for COVID-19 and history of contact with someone who had tested COVID-19 positive. Size of the household was categorized into three groups—households with < 5 members, 5–6 members and 7 + members based on the distribution of household size found in the study. Comorbidity status refers to self-reported hypertension or diabetes at the time of the survey. Percent distribution, mean, Standard Deviations (SD), and median were reported to describe the characteristics of the participants. The pooled data from the selected 11 districts were used to estimate the seroprevalence of antibodies against SARS-CoV-2 with 95% CI at the aggregate level for overall as well as by age, gender and place of residence. Appropriate sampling weights were computed to generate weighted seroprevalence. Further, the weighted seroprevalence was adjusted for the test performance considering the estimated sensitivity (92.37%) and specificity (97.90%) of the assay [24] using the formula [25]:

$${\text{Adjusted seroprevalence}} = \frac{{{\text{weighted seroprevalence}} + {\text{specificity}} - 1}}{{{\text{sensitivity}} + {\text{specificity}} - 1}}.$$

Adjusted seroprevalence was also estimated for each of the 11 districts and as well as stratified by place of residence (rural, urban), age group (5–17 years, 18 years and above) and gender (male, female). The variation in the seroprevalence by PSUs was also analysed to assess the association of seroprevalence and population size. That is, we examined whether bigger PSUs (measured through relatively higher population size) had higher seroprevalence compared to the smaller PSUs. Further, PSUs were grouped into three categories based on the distribution of seroprevalence at the PSU level. Since the overall seroprevalence in the 11 districts was 22%, we created the following three categories: (1) PSUs with zero seropositivity versus; (2) those having seropositivity up to the near average (< 20%); and (3) those PSUs with more than 20% seroprevalence. The analysis was carried out among 16,012 participants using STATA version 16.0 [26].

### Ethical Considerations

The study received ethical approval from the Institutional Ethics Review Board (IERB) of KGMU, Lucknow (1062/ethics/2020). Written informed consent was obtained from each adult participant before the interview. For participants aged 5–17 years, assent was obtained from them and written consent was also taken from the parents or the adult member who accompanied them at the study site. The study also allowed a maximum of an additional five samples from each PSU to accommodate volunteer participation. The anonymity of the respondents was maintained by labelling each participant with a unique identification code composed of the PSU number and the serial number of participants generated automatically in ODK.

## Results

Of the total 16,012 participants, 7000 (47.0%) were from urban and 9012 (53.0%) were from rural areas. While 8253 (51.2%) respondents were male, 7759 (48.8%) were female. Participants aged 5–17 years comprised 21.3% of the total sample, while those aged 18–39 years, 40–59 years and 60 + years constituted 48.6%, 27.3% and 2.9% of the sample, respectively. Of the total respondents aged 18 and above, 23.7% were engaged in either business or service, 23.0% were employed in agriculture, 31.9% were homemakers and 12.3% were unemployed. Furthermore, 10.0% of adults were smokers, and 3.7% reported any comorbidity. History of contact with a person having a confirmed case of COVID-19 was reported by 1.6% of participants (Table 1).

**Table 1:** Participant profile

Characteristics	Participants, *n* (%)
Total	16,012 (100.0)
Residence	
Urban	7000 (47.0)
Rural	9012 (53.0)
Household size	
< 5	3877 (25.1)
5–6	6227 (39.1)
7 +	5494 (33.0)
Missing	414 (2.8)
Mean household size (SD) (*n* = 15,598)	6.2 (2.7)
Age, years	
5–17 years	3493 (21.3)
18 + years	12,519 (78.7)
18–39 years	7601 (48.6)
40–59 years	4378 (27.3)
60 + years	540 (2.9)
Median age, years (25th, 75th)	30 (19, 40)
Gender	
Male	8253 (51.2)
Female	7759 (48.8)
Current occupation (aged 18 +) (*n* = 12,519)	
Professionals	696 (5.5)
Business	1936 (18.2)
Agricultural	3234 (23.0)
Housewife	4119 (31.9)
Unemployed	1334 (12.3)
Other	860 (6.5)
Missing	340 (2.8)
Government employee (among aged 18 +) (*n* = 12,519)	229 (1.6)
Working outside of home district (among aged 18 + and working) (*n* = 6726)	279 (3.9)
Work place was under containment zone/in hotspot areas in last 6 months (among aged 18 + and working) (*n* = 6726)	597 (10.8)
Travelled for work or other purposes in last 6 months (among aged 18 +) (*n* = 12,519)	
Travelled within district	2273 (16.8)
Travelled between districts	383 (2.5)
Travelled to other state or abroad	83 (0.4)
Had any comorbidity (among aged 18 +) (*n* = 12,519)	485 (3.7)
Smoking (among aged 18 +) (*n* = 12,519)	1385 (10.0)
History of COVID-19-positive of any household member in last 6 months	137 (1.2)
History of COVID-19-positive of any household member at the time of survey	29 (0.4)
History of COVID-19-positive of any person from this village/ward or neighbourhood in last 6 months	1414 (8.2)
History of contact with an individual diagnosed with COVID in last 6 months	162 (1.6)

Table 2 depicts the seroprevalence rates across the different population groups. For the 11 districts combined, the weighted seroprevalence adjusted for test performance was 22.2% [95% CI 21.5–22.9] with 30.6% seropositivity in urban areas [95% CI 29.4–31.7], and 14.7% [95% CI 13.9–15.6] in rural areas. The seropositivity was 18.4% [95% CI 17.0–19.9] among younger participants aged 5–17 years, followed by 22.5% [95% CI 21.4–23.5], 24.3% [95% CI 22.9–25.7] and 25.4% [95% CI 21.3–30.0] among participants aged 18–39 years, 40–59 years and 60 + years, respectively. No major variations in seropositivity rates were noticed between males (22.2% [95% CI 21.2–23.2]) and females (22.2% [95% CI 21.2–23.2]), and among participants belonging to different household sizes—23.3% [95% CI 21.9–24.8] in household size < 5, 22.0% [95% CI 20.9–23.2] in household size of 5–6 and 21.5% [95% CI 20.3–22.7] in household size of 7 +.

**Table 2:** Seroprevalence [95% CI] by characteristics of participants in UP

Characteristics	Participants, *n*	Seropositive participants, *n*	Unweighted seroprevalence, % [95% CI]	Weighted seroprevalence, % [95% CI]	Weighted seroprevalence adjusted for test performance^+^, % [95% CI]
Total	16,012	3332	20.8 [20.2–21.4]	22.1 [21.5–22.8]	22.2 [21.5–22.9]
Residence					
Urban	9012	1411	27.4 [26.4–28.5]	29.7 [28.7–30.7]	30.6 [29.4–31.7]
Rural	7000	1921	15.7 [14.9–16.4]	15.4 [14.7–16.2]	14.7 [13.9–15.6]
Household size					
< 5	3877	830	21.4 [20.2–22.7]	23.1 [21.8–24.5]	23.3 [21.9–24.8]
5–6	6227	1257	20.2 [19.2–21.2]	22.0 [21.0–23.0]	22.0 [20.9–23.2]
7 +	5494	1168	21.3 [20.2–22.4]	21.5 [20.4–22.6]	21.5 [20.3–22.7]
Age					
5–17 years	3493	622	17.8 [16.6–19.1]	18.7 [17.4–20.1]	18.4 [17.0–19.9]
18 + years	12519	2710	21.7 [20.9–22.4]	23.1 [22.3–23.8]	23.2 [22.4–24.0]
18–39 years	7601	1597	21.0 [20.1–21.9]	22.4 [21.5–23.3]	22.5 [21.4–23.5]
40–59 years	4378	948	22.5 [21.3–23.7]	24.1 [22.8–25.3]	24.3 [22.9–25.7]
60 + years	540	129	23.9 [20.5–27.7]	25.0 [21.3–29.2]	25.4 [21.3–30.0]
Gender					
Male	8253	1749	21.2 [20.3–22.1]	22.1 [21.2–23]	22.2 [21.2–23.2]
Female	7759	1583	20.4 [19.5–21.3]	22.2 [21.2–23.1]	22.2 [21.2–23.2]
Current occupation (aged 18 +)					
Professionals	696	529	24.0 [21.0–27.3]	25.0 [21.9–28.4]	25.4 [22.0–29.1]
Business	1936	1395	27.9 [26.0–30.0]	27.9 [26.1–29.8]	28.6 [26.6–30.6]
Agricultural	3234	2629	18.7 [17.4–20.1]	18.4 [17.1–19.9]	18.1 [16.6–19.7]
Housewife	4119	3251	21.1 [19.9–22.4]	24.1 [22.8–25.4]	24.3 [22.9–25.8]
Unemployed	1334	1027	23.0 [20.8–25.4]	23.5 [21.4–25.6]	23.7 [21.4–26.1]
Others	860	701	18.5 [16.0–21.2]	18.8 [16.2–21.6]	18.5 [15.7–21.6]
Government employee (aged 18 +)					
Yes	229	50	21.8 [17.0–27.7]	18.1 [13.5–24.0]	17.8 [12.6–24.2]
No	11,950	2597	21.7 [21.0–22.5]	23.2 [22.4–23.9]	23.3 [22.5–24.2]
Work place (aged 18 + and working)					
Working in home district	6447	1408	21.8 [17.0–27.7]	18.1 [13.5–24.0]	17.8 [12.6–24.2]
Working outside of home district	279	64	21.9 [20.9–22.9]	22.5 [21.5–23.6]	22.6 [21.5–23.8]
Work place came under containment zone/fall in hotspot areas (aged 18 +)					
Yes	597	139	23.3 [20.1–26.8]	25.6 [22.6–28.9]	26.0 [22.7–29.7]
No	5385	1149	21.3 [20.3–22.5]	21.6 [20.5–22.7]	21.6 [20.3–22.9]
Don’t know	744	184	24.7 [21.8–28.0]	24.1 [21.6–26.9]	24.4 [21.5–27.5]
Travelled within district for work or other purposes in last 6 months (aged 18 +)					
Yes	2273	498	21.9 [20.3–23.7]	21.2 [19.5–23.0]	21.1 [19.3–23.1]
No	10,246	2212	21.6 [20.8–22.4]	23.4 [22.6–24.2]	23.6 [22.7–24.5]
Travelled between districts for work or other purpose in last 6 months (aged 18 +)					
Yes	383	87	22.7 [18.8–27.2]	21.3 [17.1–26.2]	21.3 [16.6–26.6]
No	12,136	2623	21.6 [20.9–22.4]	23.1 [22.4–23.9]	23.3 [22.4–24.1]
Travelled to other state or abroad for work or other purposes in last 6 months (aged 18 +)					
Yes	83	16	19.3 [12.2–29.2]	18.6 [10.4–31.1]	18.3 [09.2–32.1]
No	12,436	2694	21.7 [21.0–22.4]	23.1 [22.3–23.8]	23.2 [22.4–24.1]
Had any comorbidity (aged 18 +)					
Yes	485	121	25.0 [21.3–29.0]	25.4 [21.7–29.6]	25.8 [21.7–30.4]
No	12,034	2589	21.5 [20.8–22.3]	23.0 [22.2–23.7]	23.1 [22.3–23.9]
Smoking (aged 18 +)					
Yes	1385	310	22.4 [20.3–24.7]	22.6 [20.4–25.0]	22.8 [20.3–25.4]
No	10,794	2337	21.7 [20.9–22.4]	23.1 [22.3–23.9]	23.3 [22.4–24.2]
History of COVID-19-positive of any household member in last 6 months					
Yes	137	46	33.6 [26.2–41.9]	38.8 [32.2–45.9]	40.6 [33.3–48.5]
No	15,461	3209	20.8 [20.1–21.4]	21.9 [21.3–22.6]	21.9 [21.2–22.7]
History of COVID-19-positive of any household member at the time of survey					
Yes	29	7	24.1 [12.0–42.7]	27.1 [17.6–39.4]	27.7 [17.1–41.3]
No	15,569	3248	20.9 [20.2–21.5]	22.1 [21.4–22.7]	22.1 [21.4–22.9]
History of COVID-19-positive of any person from this village/ward or neighbourhood in last 6 months					
Yes	1414	362	25.6 [23.4–27.9]	27.7 [25.3–30.2]	28.3 [25.7–31.1]
No	12,122	2430	20.1 [19.3–20.8]	21.1 [20.4–21.9]	21.0 [20.2–21.9]
Don’t know	2062	463	22.5 [20.7–24.3]	23.8 [22.2–25.5]	24.1 [22.3–25.9]
History of contact with any of the COVID-19-positives in last 6 months					
Yes	162	55	34.0 [27.1–41.6]	33.3 [27.8–39.2]	34.5 [28.5–41.1]
No	12,101	2372	19.6 [18.9–20.3]	20.9 [20.1–21.6]	20.8 [20.0–21.6]
Don’t know	3335	828	24.8 [23.4–26.3]	24.9 [23.6–26.2]	25.2 [23.8–26.7]

^+^adjusted for test performance (sensitivity 92.37% and specificity 97.9%)

The seropositivity rates were relatively higher (28.6% [95% CI 26.6–30.6]) among people engaged in business, followed by professionals (25.4% [95% CI 22.0–29.1]), homemakers (24.3% [95% CI 22.9–25.8]), unemployed (23.7% [95% CI 21.4–26.1]) and those who engaged in agricultural work (18.1% [95% CI 16.6–19.7]). Seroprevalence was 26.0% [95% CI 22.7–29.7] among those participants whose workplace came under a containment zone or were in a COVID-19 hotspot area compared to their counterparts (21.6% [95% CI 20.3–22.9]).

The prevalence rates were significantly higher among those who reported a family member being COVID-19-positive in the 6 months preceding the date of the survey (40.6%, 95% CI 33.3–48.5) compared to those who had no such exposure (21.9%, 95% CI 21.2–22.7). The participants who reported having had contact with a confirmed case in the past 6 months also had significantly higher seroprevalence (34.5%, 95% CI 28.5–41.1) compared to those with no contacts in the past 6 months (20.8%, 95% CI 20.0–21.6). Similarly, a person belonging to the same ward/village/neighbourhood with a confirmed COVID-19 case in the last 6 months, had higher chances of being seropositive (28.3% 95% CI 25.7–31.1) compared to their counterparts (21.0%, 95% CI 20.2–21.9) (Table 2).

**Fig. 1:**
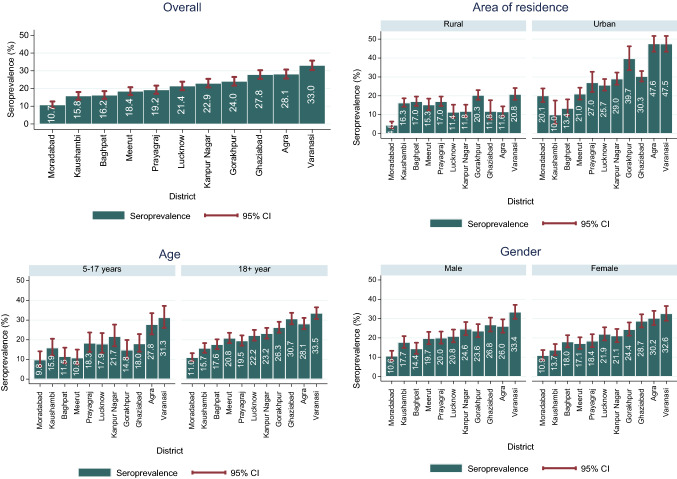
Seroprevalence (%) and 95% CI (adjusted for test performance) in 11 districts of UP, stratified by area of residence, age group and gender

There was a wide district-level differential in seropositivity ranging from 10.7% [95% CI 9.0–12.7] in Moradabad to 28.1% [95% CI 25.6–30.7] in Agra and 33.0% [95% CI 30.4–35.7] in Varanasi (Fig. 1). Seropositivity ranged from 10.0 to 47.5% in urban areas and 4.5–20.8% in rural areas across the districts. Except for Kaushambi and Baghpat districts, we found higher seropositivity in urban areas compared to rural areas. Furthermore, it was observed that in certain districts, including Varanasi, Agra, Kanpur Nagar, Prayagraj, Kaushambi and Moradabad, the seropositivity was similar among 5–17 years and 18 + years whereas in the rest of the districts the seropositivity was slightly higher among adults compared to the younger population.

**Fig. 2:**
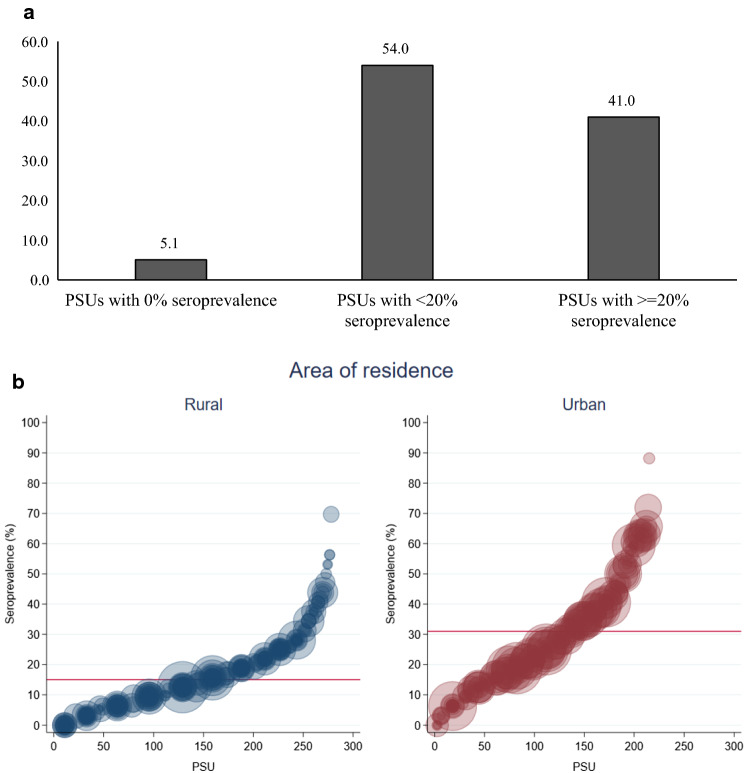
Distribution of PSUs according to seroprevalence (**a**) and seroprevalence in PSUs by area of residence (**b**), *n* = 493. Circle represents population size of the PSUs (278 Rural PSUs and 215 Urban PSus)

**Fig. 3:**
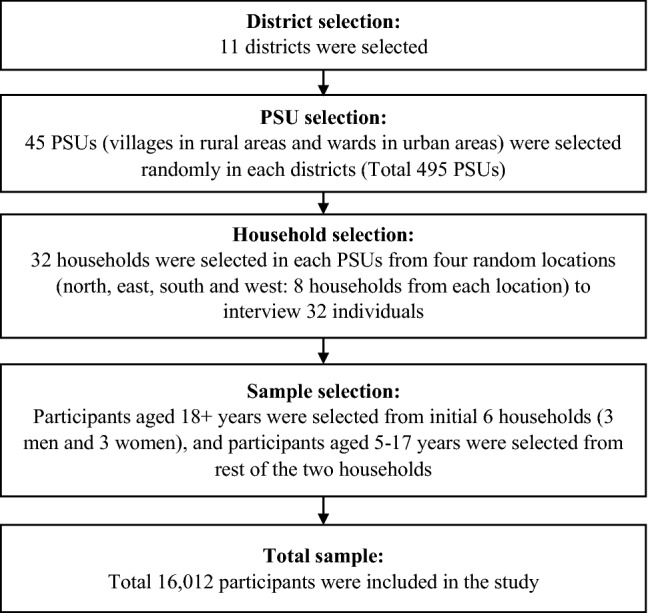
Framework of sample selection

Findings presented in Fig. 2a show that 5.1% of PSUs had zero seroprevalence whereas 54.0% and 41.0% PSUs had at least 20% or more than 20% seroprevalence, respectively. In addition, 38.6% of rural PSUs had seropositivity more than the overall level of rural seropositivity, while 47.5% of urban PSUs had seropositivity more than the overall level of urban seropositivity. Seropositivity levels did not vary by the PSU size (Fig. 2b). Table 3 presents the participant’s characteristics among the three PSU clusters with no, moderate and high seropositivity. In high seropositivity PSUs, a relatively larger proportion of participants were from urban areas (70%), engaged in business (25.0%) and their workplace fell under a containment zone (13.1%). Also, PSUs with higher seropositivity had a higher proportion of persons (10.5%) reporting a confirmed case of COVID-19 person from their community in the 6 months preceding the survey compared to PSUs with moderate seropositivity PSUs (6.8%) and PSUs with no seropositivity (1.7%).

**Table 3:** Profile of participants by PSU clusters according to seroprevalence

Characteristics	PSU clusters based on seroprevalence			*p* value
	PSUs with 0% seroprevalence (*n* = 795)	PSUs with < 20% seroprevalence (*N* = 8668)	PSUs with ≥ 20% seroprevalence (*N* = 6549)	
Residing in urban areas	13.1	30.7	70.1	0.000
Mean household size (SD)	6.2 (2.4)	6.3 (2.8)	6.0 (2.7)	0.000
Mean age, years (SD)	31.3 (15.0)	30.9 (14.7)	31.2 (14.5)	0.307
Male	52.8	51.0	51.3	0.483
Current occupation (aged 18 +)				0.000
Professionals	2.7	6.0	5.1	
Business	5.6	13.4	25.0	
Agricultural	48.2	27.5	15.0	
Housewife	29.1	32.5	31.4	
Unemployed	5.1	11.4	14.2	
Others	9.1	2.6	3.3	
Government employee (among aged 18 +)	1.2	1.5	1.8	0.072
Working in other than home district (among aged 18 + and working)	1.7	4.1	4.0	0.011
Work place came under containment zone/fall in hotspot areas in last 6 months (among aged 18 + and working)	0.1	10.1	13.1	0.000
Travelled within district for work or other purposes in last 6 months (among aged 18 +)	14.4	19.7	13.7	0.005
Travelled between districts for work or other purposes in last 6 months (among aged 18 +)	2.0	2.5	2.5	0.175
Travelled to other state or abroad for work or other purposes in last 6 months (among aged 18 +)	0.3	0.5	0.4	0.983
Had any comorbidity (among aged 18 +)	0.4	3.6	4.1	0.000
Smoking (among aged 18 +)	6.2	10.5	9.9	0.021
History of COVID-19-positive of any household member in last 6 months	0.3	1.0	1.5	0.052
History of COVID-19-positive of any household member at the time of survey	0.3	0.5	0.3	0.391
History of COVID-19-positive of any person from this village/ward or neighbourhood in last 6 months	1.7	6.8	10.5	0.000
History of contact with any of the COVID-19-positives in last 6 months	0.1	0.9	2.6	0.000

## Discussion

Our study was the first seroepidemiological study in UP which provides district-level estimates of SARS-CoV-2 antibodies in high-burden districts of UP. We found that 22.2% of the population, equating to 10.4 million people in 11 districts, was exposed to SARS-CoV-2 infection by early September 2020. We also identified significant heterogeneity across the state with seroprevalence ranging from 10.7 to 33.0%.

A few other states in India, and several other countries, have conducted population-based seroprevalence studies to provide information about the situation of the epidemic from time to time. For example, the results of analysis from 38 countries showed seroprevalence ranging from 0.1 to 38.1% by mid-September 2020 [27]. Similarly, studies from urban cities across India (Indore, Haryana, Chennai, Delhi, and Pune) had shown that 8–52% of the population was infected in the initial 6–8 months of the onset of the COVID-19 pandemic [28–32].

The three rounds of national serosurvey in India between May and December 2020 showed an increasing seropositivity trend. The second national serosurveillance study, conducted by ICMR at the same time period as this study, showed only 6.6% of India’s population aged 10 + years were exposed to SARS-CoV-2 by mid-September 2020. In the nine districts of UP that were included in the national seroprevalence survey, a low prevalence was detected, ranging from 1% in Aurayia to 13% in Mau district, suggesting that these districts were in the early phases of the epidemic [12]. In contrast, our study revealed a higher seroprevalence in the 11 districts in which our study was implemented, indicating a more advanced stage of the epidemic by mid-September 2020. The differences in the estimates provided by the ICMR study and our study are likely to be attributed to the differences in the sampling design. Our study included a larger sample (1440) per district to provide representative district-level estimates, whereas the ICMR study was designed to provide national estimate covering 400 samples from selected districts. Also, the higher sample size in this study allowed us to provide representative district-level rural–urban estimates, which was not the purpose of the national serosurveillance study.

The results also confirmed a distinctly higher exposure to SARS-CoV-2 in urban areas compared to rural areas until September 2020. Other studies conducted in India also reported lower seroprevalence in rural areas (5.2%) compared to urban areas (9.0% in urban non-slum and 16.9% in urban slum areas) [12]. Moreover, serosurveyes in other Indian cities estimated seropositivity as high as 51.3% in Pune city, 54.1% and 16.1% in urban slum and non-slum areas in Mumbai city, 23.2% in Ahmedabad city and 22.9% in Delhi [12, 16–18]. Urban areas are densely populated with less well ventilated and compact houses and have higher population mobility as compared to rural areas, both factors likely contributing to higher transmissibility [33]. The higher proportion of the unexposed population (78%) in these 11 districts, necessitates the need for continuous monitoring and focused testing.

The present study was uniquely designed to provide a representative estimate of seropositivity by gender and age. Based on the overall adjusted seroprevalence, the younger population (age 5–17 years) had less exposure to the SARS-CoV-2 infection compared to the adult or elderly population. We found no significant difference in seropositivity between men and women. A similar pattern was also observed at the national level [12] wherein men and women had similar seroprevalence and adults and elderly had relatively higher (though not statistically significant) seroprevalence. There is limited availability of sex and age disaggregated data in India, thus hampering analysis of gendered implications of COVID-19. Evidence on the gender and age disaggregated COVID-19 was largely mixed in nature with few studies showing a greater occurrence of infection in men while others found a similar distribution of infection by gender with some variation across age group [4, 11, 12].

Furthermore, the study found large geographical heterogeneity in seroprevalence across the PSUs covered in the study. The PSU-level seroprevalence ranged from 0 to 88.2% in urban areas and 0 to 69.7% in rural areas. We also assessed the PSU-level heterogeneity in seropositivity to understand the differential in the characteristic of the geographies with no-seropositivity versus medium or high level of seropositivity. Clearly, the PSUs with high seropositivity had a larger proportion of the population with a larger exposure to COVID-19 compared to moderate and no-seropositivity PSUs. These findings bring evidence for the need to adopt a differential geography-specific containment, surveillance and treatment strategy. While a robust surveillance method and high testing levels are important for geographies with lower seropositivity, it is also important to ensure that these areas are suitably equipped with proper treatment facilities. Due to their heightened vulnerability to any potential escalation in cases, it is vital to ensure service readiness to avoid sudden overstraining of the healthcare system. On the other hand, in high prevalence geographies, there is a need to continue priority testing of symptomatic individuals and patients with co-morbidities and contacts of confirmed cases. Disease management strategies in such regions can also serve as a template for newer geographies of focus, where the virus spread is still in a relatively nascent stage. For example, if facility-based clinical management is only required for a select demographic group or people with co-morbidities, then these learnings should be utilised to further inform home treatment policies and protocols. This could ease pressure on health service system resources so that critical cases could be managed properly.

The present serosurveillance study has certain limitations. Although the study provides district-level representative estimates, the seroprevalence estimates cannot be generalized for the whole state as the 11 districts were not randomly selected. Second, similar to other studies, the prevalence estimates can be affected by the test specificity. Third, since the study included only one respondent from the selected household, the effect of household size on seropositivity cannot be ascertained. Lastly, we may have missed some people with very recent infection as their IgG might not have developed during the acute phase of the disease at the time of the survey. However, despite these limitations, the first seroprevalence study in the state provides important information about the population already exposed to SARS-CoV-2 infection versus those susceptible to infection. A repeat cross-sectional survey may help to shed light on the evolving trajectory of the virus, as well as the effectiveness of the existing disease monitoring and management systems. Along with strengthening sentinel surveillance, as the risk of COVID-19 changes over time, such exercises will aid the state and local administration in formulating disease management and containment planning strategies to the best needs of the local populace. Furthermore, the findings are of considerable assistance as the state works to resume service in other priority health domains to pre-COVID levels. A repeat seroprevalence study among COVID positive cases may also be able to provide insight about the duration until which antibodies persist.

## Conclusion

Since the onset of the COVID-19 pandemic, India has witnessed a concentration of a higher number of positive cases in certain geographies. Population-based serosurveys aid not only in identifying the proportion of population infected but also as an estimate of the remaining susceptible population, thus providing valuable information for future planning and mitigation efforts to contain further spread. We found that nearly one in five individuals aged five and above were exposed to SARS-CoV-2 infection in UP by mid-September 2020 when the state witnessed its first peak. The findings also indicate that a substantial proportion of the population in 11 districts remain susceptible. Therefore, targeted contact tracing and testing remain critical to control the transmission of the virus. Moreover, the health facilities need to be continuously monitored to ensure that the facilities providing COVID management are prepared. Recognizing the fact that India, as well as UP, is currently witnessing a second wave of the epidemic, another round of seroprevalence survey and/or a seroconversion survey among already infected individuals might additionally give insights into disease epidemiology. Ascertaining the geographies wherein susceptibility among the general populace is greater would help in informing strategic allocation of resources by the government. The study also offers an example of a plausible methodology to conduct the state-specific serosurveys to those states which are yet to conduct such state-level serosurveys.

## Appendix

See Table 4 and Fig. 3.

**Table 4:** Weighted seroprevalence [95% CI] adjusted for test performance by place of residence, age group and gender in 11 districts of UP

	Overall	Place of residence		Age group		Gender	
		Urban	Rural	5–17 years	18 + years	Male	Female
Agra	28.1 [25.6–30.7]	47.6 [43.4–51.7]	11.6 [9.3–14.4]	27.8 [22.7–33.5]	28.1 [25.4–31.1]	26.0 [22.7–29.6]	30.2 [26.6–34]
Baghpat	16.2 [14.2–18.5]	13.4 [9.6–18.1]	17.0 [14.7–19.6]	11.5 [8–16]	17.6 [15.2–20.2]	14.4 [11.7–17.5]	18.0 [15.1–21.4]
Ghaziabad	27.8 [25.4–30.3]	30.3 [27.6–33.1]	11.8 [7.5–17.7]	18.0 [13.9–22.9]	30.7 [27.9–33.7]	26.8 [23.3–30.5]	28.7 [25.4–32.2]
Gorakhpur	24.0 [21.6–26.5]	39.7 [33.6–46.2]	20.3 [17.9–23]	14.8 [10.7–19.9]	26.3 [23.6–29.1]	23.6 [20.4–27.1]	24.4 [21.1–28]
Kanpur nagar	22.9 [20.6–25.4]	29.0 [25.9–32.3]	11.8 [8.9–15.3]	21.7 [16.7–27.7]	23.2 [20.6–26]	24.6 [21.4–28.2]	21.1 [17.8–24.6]
Kaushambi	15.8 [13.7–18]	10.0 [5.1–17.5]	16.3 [14.2–18.7]	15.9 [12–20.5]	15.7 [13.4–18.3]	17.7 [14.8–20.9]	13.7 [11–16.8]
Lucknow	21.4 [19.1–23.8]	25.7 [22.8–28.9]	11.4 [8.4–15.3]	17.9 [13.4–23.4]	22.2 [19.6–25]	20.8 [17.7–24.3]	21.9 [18.7–25.5]
Meerut	18.4 [16.3–20.7]	21.0 [18–24.3]	15.3 [12.4–18.5]	10.8 [7.5–15]	20.8 [18.3–23.5]	19.7 [16.7–23.1]	17.1 [14.3–20.3]
Moradabad	10.7 [9–12.7]	20.1 [16.7–23.9]	4.5 [2.9–6.4]	9.8 [6.5–14.1]	11.0 [9.1–13.2]	10.6 [8.3–13.3]	10.9 [8.5–13.7]
Prayagraj	19.2 [17–21.6]	27.0 [22–32.7]	17.0 [14.6–19.6]	18.3 [13.8–23.7]	19.5 [17–22.2]	20.0 [17–23.3]	18.4 [15.2–21.9]
Varanasi	33.0 [30.4–35.7]	47.5 [43.4–51.7]	20.8 [17.9–24.1]	31.3 [26–37.2]	33.5 [30.6–36.5]	33.4 [29.9–37.1]	32.6 [28.9–36.5]

## Declarations

### Research data

The datasets used and/or analysed during the current study are available from the corresponding author after the GoUP permission on reasonable request.
